# Evaluation of the sustainable development of logistics based on local characteristics: Matching logistics architecture with resources

**DOI:** 10.1371/journal.pone.0307078

**Published:** 2024-10-15

**Authors:** Xin Wen, Yan Liu

**Affiliations:** 1 School of Management, Shenyang University of Technology, Shenyang City, Liaoning Province, China; 2 School of Economics and Management, Shanghai Polytechnic University, Pudong District, Shanghai City, China; National University of Sciences and Technology, PAKISTAN

## Abstract

This paper sets out to investigate the alignment between the logistics architecture and resources across China’s nine logistics regions, while also examining the individual advantages these regions possess. With the goal of establishing an evaluative framework for sustainable logistics development, the research introduces the principle of logistics equilibrium to augment traditional evaluation metrics, thus forming an assessment index system designed to quantify the congruence between logistics architecture and resources.Utilizing a democratic evaluation approach that emphasizes individual regional advantages, and leveraging empirical data from the decade spanning 2011 to 2020, this paper reveals a general upward trajectory in the matching level of logistics resources to architecture albeit with notable regional disparities. It is observed that while certain areas have made significant strides, others lag, underscoring the varied pace of development among different logistics regions.The research also brings to light the distinct advantage characteristics that emerge as each region evolves, suggesting that these attributes can be harnessed to foster a more environmentally conscious and forward-thinking approach to logistics. By implementing a democratic evaluation to rank these regions, this paper aims to identify exemplars that are not only recognized for their achievements but also serve as models for the formulation of green and sustainable logistics strategies tailored to each region’s specific needs and potential.

## I. Introduction

In the contemporary backdrop of globalization and regional economic integration, logistics stands as an indispensable link between production and consumption, playing an integral role in the sustainable development of the economy. The establishment of a green and efficient logistics system is paramount, as it can enhance the efficiency of resource utilization, minimize waste, and is particularly crucial for regions with constrained resources. Furthermore, it can accelerate the movement of goods, reduce logistics costs, and increase production efficiency, thereby stimulating sustainable economic growth, elevating the quality of economic development, and strengthening regional competitiveness [1].

Despite the pivotal role of the logistics industry in economic development, significant changes in China’s logistics industry, including its development model, architecture, and resources, have led to persistent challenges such as low logistics resource utilization efficiency, low levels of sustainable development matching between logistics resources and architecture, redundant logistics functions across regions, and a lack of distinct competitive advantages [2]. On one hand, some regions have not fully leveraged their resource advantages to establish a logistics architecture that aligns with local characteristics. On the other hand, the logistics architecture established in certain regions are highly similar to those of neighboring areas, leading to redundant resource allocation. Consequently, there are notable differences across regions in how they utilize local logistics resources, build a corresponding logistics architecture, and achieve green and sustainable development in logistics. These discrepancies may impact the matching level between logistics resources and architecture, necessitating an analysis based on the unique resource advantages of each region to formulate targeted optimization strategies.

The formation of a matching relationship between logistics resources and architecture must be grounded in the region’s resource endowments. As the resource-based theory posits, competitive advantages stem from unique and heterogeneous resources [3]. Similarly, the design of a logistics architecture should take into account the region’s geographical conditions, resource endowments, and industrial structure to ensure that various logistics resources are compatible with the designed logistics structure. The theory of competitive excellence also emphasizes the importance of capitalizing on individual strengths, avoiding cutthroat competition, and striving for a "competitive excellence" ecosystem [4]. Therefore, evaluating the matching level of logistics resources and structure based on individual characteristic advantages can more effectively reveal the advantageous or disadvantageous factors that influence their compatibility.

Individual characteristic advantages are defined as the unique advantages a subject possesses when a particular distribution of indicator weights results in a superior evaluation outcome compared to any other weighting scheme. When each subject selects its own characteristic advantage weights based on its perspective and evaluates all subjects accordingly, it results in personalized traits and relative advantages [5]. Since the determination of individual characteristic advantage weights is free from human intervention, this evaluation method is considered objective.

Current research predominantly focuses on the operational efficiency of green logistics, its relationship with economic development, and the efficient utilization of logistics resources. However, there is a need for further exploration from a regional perspective on the critical issues of green logistics construction, operation, and sustainable development evaluation. This includes assessing whether the individual characteristic advantages of regional logistics resources are being fully utilized and whether the established logistics architecture is in harmony with the available resources. Additionally, when selecting evaluation indicators, it is essential to consider the relevant influencing factors and construct an appropriate evaluation indicator system.

This study specifically aims to address the question of how to construct a green and sustainable logistics architecture based on regional resource advantages. By identifying the individual characteristic advantages of a region’s logistics and establishing a scientifically sound evaluation method for the matching relationship between logistics architecture and resources, this research provides a basis for enhancing their compatibility. The study employs an indicator system to measure the matching level of logistics structure and resources, utilizes the entropy method to determine the weights of each indicator, applies the TOPSIS method to calculate the matching level, and employs a goal programming model to identify individual characteristic advantages. Through an empirical analysis of the matching between logistics architecture and resources in China’s nine major logistics regions from 2011 to 2020, the effectiveness of the proposed evaluation method is validated. The study also reveals that improving the matching level of logistics architecture and resources requires leveraging individual characteristic advantages and achieving balanced development across four dimensions. This research introduces a novel evaluation method for the matching level of logistics architecture and resources based on individual characteristic advantages, offering a scientific basis for policymakers and contributing to the green and sustainable development of regional logistics.

The paper is structured as follows: Section II will conduct a literature review. Section III will provide a detailed introduction to the research methods employed. Section IV will present the evaluation indicators and data sources. Section V will conduct an empirical analysis. Finally, Section VI will summarize the research findings.

## II. Literature review and hypotheses development

Green logistics is defined as the practice of integrating environmental considerations into the logistics process, with the goal of minimizing the ecological impact of logistics activities while maximizing efficiency and effectiveness[6]. It involves the study and implementation of efficient and sustainable supply chain management practices. Scholars have delved into various aspects of green logistics, with extensive research indicating that a multitude of issues surrounding green logistics deserve ongoing attention [7]. The reduction of logistics costs and the enhancement of operational efficiency have emerged as fundamental objectives within the logistics industry. However, the compartmentalization of the logistics sector has accustomed people to assessing the level of green logistics from a singular viewpoint, such as the optimization of transportation routes, collaboration across different departments, and the establishment of green environmental policies. Macroscopically, scholars have also conducted multifaceted studies on the evaluation of the green development of the logistics industry. For instance, Wang L (2021) quantified the influence of the coupling and coordination between the logistics and financial sectors on the carbon emissions of the logistics industry [8]. Jia K (2021) employed the DEA-BCC model to assess the green logistics standards across various nations [9]. Gan W et al. (2022) highlighted that the conservation of resources and the logistics industry’s transition from a low-level, extensive growth model to a high-quality development phase represent a novel stage in the evolution of green logistics, one that emphasizes recycling and efficiency [10]. Consequently, it is imperative to concentrate on the pivotal trajectory of green logistics development, which involves identifying regional resource advantages, constructing a sustainable logistics structure that aligns with these strengths, and thereby stimulating the green growth of regional logistics.

Upon deeper examination of the scholarly outcomes concerning the optimized utilization of logistics resources, it becomes evident that the research emphases within this domain differ significantly. This variation is attributable to the distinct nature of logistics resource endowments and the diverse approaches to their application. Ghatak RR (2023) provided a new perspective and approach for understanding the barriers to logistics resource integration in omnichannel retail by integrating the ISM and fuzzy MICMAC methods [11]. Kolev D et al. (2022) analyzed the application of ERP systems in logistics service departments and their impact on enterprise logistics management through case studies, emphasizing the importance of integrating ERP systems with enterprise logistics resources [12]. Kadlubek M et al. (2022) explored the application of intelligent transportation systems in highway freight enterprises, aiming to enhance their customer service capabilities by integrating logistics resources [13]. Daron M (2022) pointed out through a case study that appropriate logistics resource planning is a key factor affecting process quality and efficiency and used FlexSim software for simulation [14]. Breitbarth E et al. (2021) studied the collaboration between logistics service providers, retailers, and public authorities during disease outbreaks to develop a vehicle routing problem (CVRP) model with capacity and combined it with a k-means clustering algorithm to optimize the use of logistics resources for home delivery services to vulnerable populations [15]. In the study of matching production to logistics resources. An Z et al. (2024) utilized the fsQCA approach to perform a multifactorial configuration analysis. Their research underscored the pivotal role of investments in logistics resources, including infrastructure, in the harmonious development trajectory that aims to foster regional economic progress and bridge the gap in regional development disparities [16]. In terms of logistics resource allocation, Lin W et al. (2022) started from the input‒output of logistics resources, constructed a system to evaluatethe level of logistics resource allocation, and conducted an empirical analysis [17]. Zhang et al. (2021) focused on cloud logistics resources and constructed a cloud logistics resource allocation model based on spectral machine learning in a big data environment [18]. Deng S et al. (2020) developed and validated a range of logistics resource-sharing initiatives aimed at optimizing multi-center collaborative logistics distribution networks through a resource-sharing framework. Additionally, they introduced the AGPSO algorithm, which has been proven to significantly improve the efficiency of resource utilization [19]. In terms of logistics resource integration and optimization, Suo L et al. (2021) noted the types of risks faced by logistics resource integration and introduced the fuzzy comprehensive evaluation method to quantify the integration risks, establishing a service integrator model for rural end logistics resource integration and optimization [20].

The studies previously mentioned, which pertain to the efficient utilization of logistics resources, provide valuable insights for the thematic exploration of this research. Yet, regardless of the methodology—be it the application of singular or multiple indicators, or even the analysis of input-output efficiency—these approaches offer only a glimpse into the utilization efficiency of logistics resources and the establishment of logistics systems. They fall short of fully delineating the intricate relationship with the green development of regional logistics and its profound implications.

In view of these, the following hypotheses were proposed:

**H1**. Building a logistics architecture that leverages a region’s resource characteristics can significantly foster its green development.

With the swift economic growth in China and the evolving market demands, there has been a pronounced emphasis on refining the regional economic architecture, transitioning to new growth models, and bolstering regional inter-connectivity. Regions with a well-structured logistics framework and efficient utilization of their resource wealth can effectively mitigate environmental pollution and steer towards a trajectory of sustainable logistics development.

Optimizing logistics networks and the allocation of resources can curtail superfluous transportation and storage needs, subsequently reducing energy use and the emission of greenhouse gases [21–22]. A strategic alignment of logistics infrastructure with available resources can minimize the excessive use of packaging and material wastage, thereby advocating for a circular economy and the incorporation of sustainable materials [23]. The deployment of sophisticated logistics planning tools and algorithms can streamline transportation routes, minimize empty miles traveled, enhance fuel efficiency, and consequently shrink the carbon footprint [24]. By aligning logistics resources effectively, such as the adoption of electric or hybrid vehicles, the reliance on fossil fuels can be diminished, leading to lower emissions [25]. Furthermore, a well-optimized logistics structure and resource utilization empower regions to navigate market volatility and potential supply chain disruptions more resiliently, reinforcing the sustainability of the logistics system as a whole.

**H2**. Different logistics regions possess local characteristics, and the elements of these strengths significantly influence the assessment of how well the logistics system aligns with logistics resources.

Recognizing and capitalizing on the distinctive attributes of various logistics regions is essential for enhancing the efficiency, sustainability, and robustness of the logistics system [26]. Each region’s advantages are derived from its geographical position, infrastructure, regulatory framework, and economic landscape. These benefits might encompass high logistics demand, a well-structured industry, advantageous geographical positioning and network design, and expedited logistics services. These factors are interconnected, and their harmonized growth can foster the sustainable and resilient development of the logistics system [27–30]. It is imperative to align the logistics system with the region’s unique strengths, and the efficacy of this alignment is critical for shaping development strategies [31]. For instance, regions with advanced port facilities might focus more on maritime logistics than on land transportation. Regions endowed with abundant renewable energy resources might opt to deploy electric vehicle fleets for logistics, thus reducing emissions. Moreover, the alignment of the logistics system with regional resource characteristics can substantially influence economic performance [32]. Streamlined logistics can decrease costs, enhance market accessibility, and stimulate economic growth. A logistics system that is well-aligned with regional strengths is likely to be more resilient to disruptions. For example, regions with diverse transportation modes can more effectively manage disruptions in one mode by leveraging others. Consequently, the elements of individual advantage characteristics are of great significance and value in evaluating the alignment between the logistics architecture and logistics resources. They also offer a strategic foundation for the green and sustainable progression of regional logistics.

**H3**. Attaining the objective of logistics equilibrium is a crucial pathway to fostering the green and sustainable growth of regional logistics.

Logistics equilibrium represents a pivotal concept within the logistics discipline, addressing the equilibrium of supply and demand within the logistics system. It mandates that logistics supply and demand are closely matched in terms of quantity, structure, time, and spatial distribution across every tier of the supply and industrial chain [33]. This balance is essential for enhancing the operational efficiency of the logistics system, minimizing costs, and bolstering the supply chain’s stability and agility. In the context of regional logistics development, a well-balanced logistics system can optimize the utilization of logistics resources, elevate transportation efficiency, and consequently mitigate environmental pollution. Additionally, logistics equilibrium contributes to curtailing transportation expenses and amplifying the economic profitability of logistics enterprises. It facilitates the inter-regional exchange of resources and economic collaboration, thereby fostering coordinated regional development. A balanced logistics system is also better equipped to manage contingencies, fortifying the supply chain’s robustness and adaptability. Moreover, it bolsters the punctuality and dependability of logistics services, aligning with the multifaceted demands of customers [34].

In conclusion, while many studies have established the intimate link between logistics resources and architecture, and have deliberated on the elements influencing the green and sustainable progression of regional logistics, along with the criteria for assessment, there remains a pressing need to approach the subject from the vantage point of regional logistics’ individual characteristics. This approach entails a thorough exploration and appraisal of the congruence between logistics resources and architecture, followed by the formulation of targeted development strategies. The architecture of a logistics system must be predicated on the unique characteristics inherent to the region in question. Given that these individual characteristics can vary significantly across different regions and even evolve within the same region over various phases of development, it becomes imperative to evaluate the alignment between the logistics architecture and its resources through the lens of these individual characteristics. Such an evaluation is not only academically enriching but also practically instrumental in charting the trajectory of regional logistics towards a future characterized by environmental sustainability, operational excellence, and enduring growth.This paper, grounded in the theory of logistics equilibrium, endeavors to quantify the alignment of regional logistics resources with their architectural configurations. It employs an individual characteristics identification model to assess the degree of this alignment, thereby providing a foundational basis for strategic development across different regions. This approach serves as a critical practical instrument, aimed at propelling regional logistics towards a path of ecological harmony, superior quality, and sustainable evolution.

## III. Research methods

This paper commences with an evaluation of the alignment between logistics resources and their structural framework, for which an index system is meticulously developed. The entropy value method is utilized to ascertain the weightings of the indices, while TOPSIS is applied to compute the level of alignment.

Subsequently, the study evaluates the congruence between logistics resources and their structure based on distinctive competitive traits. The evaluation process is delineated as follows: Initially, a model for identifying the individual characteristics is established. Thereafter, the alignment level is appraised using these characteristics, followed by a democratic assessment of the alignment level grounded in these unique characteristics.

### A. Measurement of matching level

#### 1) Measurement indicator system

Integrating the concept of logistics equilibrium theory [35], and building upon the substance of this discourse along with the findings from preceding studies [36–38], this paper has crafted a measurement index system. This system is tailored to align the architecture of logistics with its resource components. A detailed elucidation of each index is presented in Table 1.

**Table 1:** Measurement index of logistics architecture and logistics resource matches.

First-level indicator	Second-level indicator	Third-level indicator	Unit
Quantity matching	Logistics resources quantity demand	Total social logistics volume	Trillions of yuan
	Logistics resources quantity supply	Freight turnover	Billions of tons per kilometer
Structure matching	Logistics resources structure demand	Secondary industry value-added index	%
	Logistics resources structure supply	Growth rate in the installation quantity of industrial robots	%
Space matching	Logistics resources space demand	Density of development zones at or above the provincial level	no./kilometer ^2^
		Permanent population density	person/kilometer ^2^
	Logistics resources space supply	Density of scale above logistics parks	no./kilometer ^2^
		Density of public logistics facilities	kilometer/kilometer^2^
Time matching	Logistics resources planned time	Logistics efficiency index	/

*Quantity matching indicators*. Quantity matching pertains to the discrepancy between the actual quantity of resources needed for logistics within a region and the quantity that is feasibly available—essentially, the gap between the supply and demand sides of logistics resources [39]. The aggregate volume of social logistics denotes the cumulative worth of goods that transition from the procurement domain to the reception zone, marking their initial entry into the domestic consumption sphere. This metric stands as a pivotal gauge for assessing the magnitude and economic vibrancy of a nation’s or region’s logistics sector. It encapsulates the comprehensive movement of goods throughout the supply chain, thereby rendering the aggregate volume of social logistics a pertinent measure for the demand on logistics resources.

Freight turnover is a crucial metric for gauging the volume of transportation work, defined as the product of the cargo weight transported by vehicles over a specific timeframe and the distance covered. This measure reflects the overall service output delivered by the transportation sector and serves as the primary benchmark for the output of transportation enterprises. Consequently, freight turnover is a pertinent indicator for assessing the supply capacity of logistics resources. It encapsulates the efficiency and extent of freight services, offering a comprehensive reflection of the transportation sector’s contribution to logistics operations.

*Structure matching indicators*. Structure matching addresses the disparity between the actual proportion of various logistics resources and the ideal ratio demanded by a region’s logistics requirements [40]. As a fundamental sector of the economy, the logistics industry provides critical support to the circulation and growth of primary, secondary, and tertiary sectors. The planning and development of regional logistics must be conducive to the stability of the regional economy’s key industries, ensuring that the allocation of logistics resources aligns with the developmental needs and distinctive features of the region’s industrial framework. The value-added index of the secondary industry serves as a significant metric for quantifying the economic value generated by sectors such as manufacturing, mining, and construction. This index, therefore, mirrors the variances and requisites within the regional industrial structure, highlighting the importance of structural congruence in logistics resource allocation to support the economic endeavors effectively.

The high-quality development of the secondary industry is intricately linked to logistics automation, a pivotal element in its advancement. The integration of state-of-the-art technologies and machinery, such as automated sorting systems and smart warehousing solutions, can markedly enhance the production efficiency of secondary industries, including manufacturing. Furthermore, logistics automation contributes to the reduction of overall operational expenses. Logistics automation acts as a dynamic accelerant for the secondary industry’s growth and is equally significant in facilitating its transformation and modernization. As technological advancements continue apace, the role of industrial robots in logistics automation becomes increasingly prominent, with their application in the logistics sector expanding rapidly. This trend is a driving force behind decreased costs, heightened efficiency, and continuous refinement within the secondary industry.

Adhering to the principles of indicator construction, the paper selects the growth rate of industrial robot installations as an indicator of the supply capacity within the logistics resource structure. This metric captures the essence of technological progress and its impact on the logistics and, by extension, the secondary industry’s development trajectory.

*Space matching indicators*. Space matching refers to whether the spatial layout requirements of the logistics resources required in the region are consistent with the spatial layout of the logistics resources in reality [41]. The spatial demand for logistics resources mainly manifests in two aspects: industrial logistics space demand and living logistics space demand. Following the principle of indicator construction, the density of development zones at or above the provincial level, the density of permanent residents, the density of logistics parks above a certain scale, and the density of public logistics facilities can be used as indicators of the spatial demand for logistics resources. The calculation method for the density (*D*) of public logistics facilities is as follows:

$$D=\frac{L_{1}+L_{2}+L_{3}+L_{4}}{S}$$ (1)

where the highway mileage is *L*_*1*_, the railway operating mileage is *L*_*2*_, the total length of the air and mail routes is *L*_*3*_, the inland waterway mileage is *L*_*4*_, and the urban built-up area is *S*.

*Time matching indicators*. Time matching is determined by the capacity of a region’s logistics resources to fulfill the demand within the stipulated timeframe, which underscores the timeliness and operational efficiency of the logistics systems in place [42]. This concept is quantified through the logistics timeliness index (*LTI*). The *LTI* is defined as the ratio of the total volume of freight transported by each transportation mode at its scheduled times to the cumulative total freight volume.

Assuming that the on-time rate of highway logistics is *TR*_*1*_, that of railways is *TR*_*2*_, that of civil aviation is *TR*_*3*_, that of inland waterways is *TR*_*4*_, that of highway freight volume is *FV*_*1*_, that of railways is *FV*_*2*_, that of civil aviation is *FV*_*3*_, that of waterways is *FV*_*4*_, and that of total freight volume is *FV*, then

$$LTI=\frac{TR_{1}FV_{1}+TR_{2}FV_{2}+TR_{3}FV_{3}+TR_{4}FV_{4}}{FV}$$ (2)

#### 2) Determining the indicator weights

The indices within the measurement index system designed to ascertain the degree of matching are quantitative in nature. A significant advantage of employing the entropy method is its robust objectivity, which remains unaffected by subjective biases and adeptly captures the variations among different indicators [43]. Furthermore, the entropy method is adept at managing substantial volumes of intricate data, making it particularly well-suited for complex decision-making scenarios involving multiple indicators. Consequently, the entropy method is leveraged to ascertain the weights of each indicator, with the fundamental calculation steps outlined as follows:

(1) Data standardization involves the elimination of inherent units of measurement from the original data, converting it into a dimensionless format that facilitates comparison across different parameters. The minimum–maximum normalization method is employed for this standardization process, which scales the data to a common range. The formula for this method is as follows:

$$Z=\frac{\left(X-X_{\min}\right)}{\left(X_{\max}-X_{\min}\right)}$$ (3)

In this context, *Z* represents the value after normalization, *X* is the original data value, *X*_min_ is the minimum value within the data set, and *X*_max_ is the maximum value within the data set. The process ensures that all data points are scaled to a common scale, typically between 0 and 1, facilitating equitable comparison and analysis.

(2) The calculation of the indicator’s probability distribution involves determining the proportion of each indicator’s value within the entire data set. The formula for this calculation is as follows:

$$P_{ij}=\frac{X_{ij}}{\sum_{i=1}^{m}X_{ij}}$$ (4)

where, *m* is the number of samples, and *X*_*ij*_ is the value of the *i*-th sample under the *j*-th indicator.

(3) The entropy value is derived from the probability distribution to ascertain the entropy of each indicator. An indicator with a higher entropy value exhibits a lesser degree of dispersion, implying a more uniform distribution of values and thus a reduced impact on the overall evaluation. The formula for this calculation is as follows:

$$e_{j}=-k\sum_{i=1}^{m}P_{ij}\ln\;P_{ij}$$ (5)

where, *k* is a constant, usually taken as $k=\frac{1}{\ln\;m}$.

(4) Weights for the indicators are assigned by taking into account the magnitude of their entropy values. The entropy of an indicator inversely influences its weight, meaning that higher entropy results in a lower weight. The formula for determining these weights is as follows:

$$w_{j}=\frac{1-e_{j}}{\sum_{i=1}^{n}\left(1-e_{i}\right)}$$ (6)

where, *n* is the number of indicators.

#### 3) Calculating the match level

The TOPSIS method is favored for calculating the match level due to its intuitive and logical approach, which effectively harnesses data to yield more rational evaluation outcomes [44]. This method is particularly suitable for ranking problems within the realm of multi-objective decision analysis, making it apt for addressing the match-level measurement issue in this study.

TOPSIS is a comparative decision-making technique that employs the concepts of ideal and anti-ideal solutions. Its underlying concept involves identifying an ideal scenario where all metrics are optimized and a negative scenario where metrics are at their worst. The method then evaluates each alternative by calculating its geometric distance to both the ideal and negative ideal solutions, thereby quantifying the relative desirability and undesirability of each option. This approach allows for a systematic ranking of alternatives based on their closeness to the optimal state and remoteness from the least favorable state. The specific steps are as follows [45]:

(1) Decision Matrix Construction: Commence with the creation of a decision matrix that includes the performance scores of all alternative solutions under each evaluation criterion.

(2) Decision Matrix Standardization: Standardize the decision matrix to neutralize the impact of varying units and magnitudes across indicators, ensuring a dimensionless and comparable data set.

(3) Weight Determination: Assign weights to the evaluation criteria based on the decision-makers’ priorities or an objective weight assignment technique. Herein, the entropy method is utilized to determine the indicators’ weights.

(4) Weighted Standardized Decision Matrix Calculation: Multiply the standardized decision matrix by the respective weights to generate the weighted standardized decision matrix, which will be used for the subsequent comparative analysis. The calculation formula is:

$$r_{ij}=w_{j}v_{ij}$$ (7)

In this context, *v*_*ij*_ is the decision matrix, and *r*_*ij*_ is the weighted decision matrix.

(5) Establishment of Ideal Solutions: Upon examining the weighted standardized decision matrix, the optimal (best) and pessimal (worst) values for each criterion are determined. These extreme values are then utilized to construct the positive ideal solution, embodying the ideal performance across all metrics, and the negative ideal solution, which signifies the antithesis of the ideal, representing the least favorable performance on all criteria. The calculation formula is:

$$Z_{i}^{+}=(Z_{\max1},Z_{\max2},\ldots ,Z_{\max n})$$ (8)

$$Z_{i}^{-}=(Z_{\min1},Z_{\min2},\ldots ,Z_{\min n})$$ (9)

In this context, the maximum and minimum values of the *i*-th evaluation object are $Z_{i}^{+}$ and $Z_{i}^{-}$, respectively, which means the positive ideal solution for the *i*-th evaluation object is $Z_{i}^{+}$, and the negative ideal solution is $Z_{i}^{-}$.

(6) Euclidean Distance Determination: Utilize the Euclidean distance metric to ascertain the separation between each alternative solution and the conceptualized positive and negative ideal solutions within the weighted standardized decision matrix.


$$D_{i}^{+}=\sqrt{\sum_{j=1}^{m}{\left(Z_{ij\max}-Z_{ij}\right)}^{2}}$$ (10)

$$D_{i}^{-}=\sqrt{\sum_{j=1}^{m}{\left(Z_{ij\min}-Z_{ij}\right)}^{2}}$$ (11)

(7) Relative Proximity Assessment: Evaluate the relative closeness of each alternative to the positive ideal solution in comparison to the negative ideal solution. This is achieved by calculating the ratio of the distance to the negative ideal solution versus the distance to the positive ideal solution, thereby establishing a measure of relative desirability. The calculation formula is:

$$L_{i}=\frac{D_{i}^{-}}{D_{i}^{+}+D_{i}^{-}}$$ (12)

(8) Sorting: All alternative solutions are sorted by their relative proximity; the greater the relative proximity is, the better. For *L*_*i*_ values between 0 and 1, the closer the value is to 1, the closer the evaluation object is to the optimal level.

### B. Evaluation of the match level

#### 1) Choice of evaluation method

The congruence between logistics architecture and resources should be rooted in the region’s inherent resource endowment. According to the resource-based view theory, the competitive advantage of enterprises is often derived from their unique and varied resources. Consequently, the planning and design of logistics systems must take into account the region’s specific geographical conditions, resource landscape, and industrial composition. This tailored approach allows for a logistics system architecture that is congruent with the available logistics resources.

Furthermore, the theory of competitive advantage advocates for the maximization of unique traits, the avoidance of detrimental competition, and the pursuit of a development ecosystem that is underpinned by a "competitive advantage." This is to be achieved while adhering to the region’s developmental norms. Therefore, evaluating the compatibility level between logistics architecture and resources through the framework of individual characteristics can more accurately illuminate the attributes that significantly affect their alignment.

Individualized characteristics are defined as a unique strategy for the apportionment of indicator weights that, when assigned to an object under evaluation, generates more favorable outcomes than any alternative distribution scheme. As such, an evaluated object is deemed to possess individualized characteristics under this particular weight distribution framework. Each evaluated object, when it adopts its own individualized characteristic as the weighting scheme and applies this scheme for the assessment of all objects, can ascertain both its own individualized and relative characteristics [46].

The process of determining the weights associated with individualized characteristics is automated, eliminating the need for subjective human input, which endows this evaluation technique with a high degree of objectivity. The evaluation process of this method is as follows [47].

#### 2) Model for identifying individualized characteristics

*n* is the number of evaluation objects, and *m* is the number of evaluation indicators. For the evaluation problem of *n* evaluation objects based on *m* evaluation indicators, the basic idea of the identification model is as follows: under a certain distance sense, starting from the perspective that is most advantageous to the *i*-th evaluated object, weights *w*_*ij*_ are assigned to the various indicators of the *i*-th evaluated object $\bar{x_{i}}$. The structure of this vector of weight coefficients can then reflect the individual characteristics of the *i*-th evaluated object. Based on this, a goal planning model is constructed [48]:

$$\underset{w}{\min}\left\{d_{i}^{2}(\bar{x_{i}},{\bar{x}}^{*})\right\}=\underset{w}{\min}\left\{{\sum_{j=1}^{m}w_{ij}^{2}|x_{j}^{*}-x_{ij}|}^{2}\right\},$$

$$\begin{array}{l} s.t.\sum_{i=1}^{m}w_{ij}\geq 0,\\ i=1,2,\ldots ,n,\\ j=1,2,\ldots ,m \end{array}$$ (13)

where, *d*_*ik*_ is the *i*-th indicator value of the *j*-th evaluated object, $\bar{x_{i}}$ is the indicator vectovalue of *x*_*i*_, ${\bar{x}}^{*}$ is the preference outcome, *w*_*ij*_ is the *j*-th value quantity of *x*_*i*_, and $d(\bar{x_{i}},{\bar{x}}^{*})$ denotes the distance between $\bar{x_{i}}$ and ${\bar{x}}^{*}$ under the value parameter structure of *w*_*i*_. The ideal value of each evaluation indicator is the 2-norm distance between the *i*-th evaluated object and the ideal value. The smaller the value is, the closer it is to the ideal value, which is more advantageous for the ranking of the evaluated object *i*.

For an evaluated entity *i*, the weight coefficient is determined from the viewpoint that is most beneficial to it. This approach results in a unique set of weight coefficients for each evaluated object, effectively capturing their distinctive attributes. Furthermore, these coefficients are not subjectively assigned but are derived from an objective analysis of the data across all evaluated entities. Consequently, the process of identifying individualized characteristics for each evaluated object is inherently objective, ensuring a fair and data-driven evaluation.

#### 3) Match evaluation based on individualized characteristics

By applying the model referenced in Eq (13), the weight coefficients for *n* distinct sets of individualized characteristics are calculated for each of the *n* evaluated objects. Utilizing the perspective of each region and applying Eq (14), the matching levels of the remaining *n* subjects can be assessed, including the region itself. This process enables to evaluate the outcomes for each subject comprehensively as follows [49]:

dik=(xk¯,x¯*)={∑j=1m(wij*)2(xj*−xkj)2}12k=1,2,…,n (14)

where, *d*_*ik*_ is the distance between the evaluated object *k* and the ideal value from the perspective of *i*’s individual characteristics. $w_{ij}^{*}$ is the vector of individual characteristic advantages of each evaluated object. Based on this, the individual characteristics of each evaluated object can be assessed and analyzed. Since a smaller evaluation value is better, they can be sorted in ascending order. According to the "Pareto Principle" in management science, the top 10% of evaluated objects can be identified as having distinct individual characteristics; the bottom 10% lack individual characteristics; and the middle 80% of evaluated objects fall within the general range.

#### 4) Match evaluation based on democratic individualized characteristics

By applying Eq (14), the evaluation results for each evaluated object based on their own assessment of all evaluated objects can be calculated. Based on the obtained results, by averaging the values of $w_{ij}^{*}$ for each evaluation object, a new set of weight vectors can be derived. This new set of weight vectors can be seen as a collection of the "opinions" from each evaluation object, thereby achieving a democratization of the "opinions" among the evaluation objects. By using Eq (15) to summarize the opinions of each evaluated object, the democratic evaluation opinion can be derived. The democratic evaluation result, *D*_*k*_, for the individualized characteristics of evaluated object *k* is as follows [50]:

$$\begin{array}{l} D_{k}=\frac{1}{n}\sum_{i=1}^{n}d_{ik}(\bar{x_{k}},{\bar{x}}^{*})\\ k=1,2,\ldots ,n \end{array}$$ (15)

The democratic evaluation ranking results of *n* evaluated objects can be obtained by sorting them in ascending order.

## III. Empirical analysis

### A. Data acquisition and processing

A region is typically defined as a distinct geographical space, characterized by its area, shape, extent, and boundaries. Given the extensiveness of China’s territory, the country’s geographic regions are categorized in diverse ways, taking into account a range of factors including topography, climate, demographics, economy, and politics. Based on the characteristics of logistics activities and China’s relevant policy and development plans, China is divided into nine logistics regions as shown in Table 2.

**Table 2:** Main provinces and cities in China’s nine major logistics regions.

Logistics regions	Main provinces and cities
North China Logistics Region	Beijing, Tianjin, Hebei Province
Northeast Logistics Region	Heilongjiang Province, Jilin Province, Liaoning Province
Shandong Peninsula Logistics Region	Shandong Province
the Yangtze River Delta Logistics Region	Shanghai, Jiangsu, Zhejiang, Anhui provinces
Southeast Coastal Logistics Region	Fujian Province, Guangdong Province, Hainan Province
the Pearl River Delta Logistics Region	Guangzhou, Shenzhen, Foshan
Central Logistics Region	Henan Province, Hubei Province, Hunan Province, Jiangxi Province, Shaanxi Province
Northwest Logistics Region	Qinghai Province, Gansu Province, Ningxia Hui Autonomous Region
Southwest Logistics Region	Chongqing, Sichuan Province, Guizhou Province, Yunnan Province

This study draws upon pertinent data from China’s 31 provinces (excluding Hong Kong, Macao, and Taiwan), covering a period from 2011 to 2020. The primary sources of this data are the "China Statistical Yearbook," as well as the statistical yearbooks and economic and social development statistical bulletins for each of the 31 provinces. When discrepancies arise between data from various sources, the figures reported in the "China Statistical Yearbook" and on the National Bureau of Statistics’ official website are prioritized as the authoritative source. The missing original data were supplemented using interpolation methods.

To address the variations in the dimensions of the indicators, Eq (3) is implemented to standardize the initial data set, thereby facilitating a consistent and equitable comparison across all parameters.

### B. Measurement results of the match level

Based on the match level measurement indicator system constructed earlier, the entropy method is used to calculate the weights of each indicator, as shown in Table 3.

**Table 3:** Match level measurement index and weights.

First-level indicator	Second-level indicator	Third-level indicator	Unit	Index weights	Code
Quantity matching	Logistics resources quantity demand	Total social logistics volume	Trillions of yuan	13.33%	*X*_*1*_
	Logistics resources quantity supply	Freight turnover	Billions of tons per kilometer	10.50%	*X*_*2*_
Structure matching	Logistics resources structure demand	Secondary industry value-added index	%	11.30%	*X*_*3*_
	Logistics resources structure supply	Growth rate of installation quantity of industrial robots	%	9.23%	*X*_*4*_
Space matching	Logistics resources space demand	Density of development zones at or above the provincial level	no./kilometer ^2^	14.98%	*X*_*5*_
		Permanent population density	person/kilometer ^2^	9.16%	*X*_*6*_
	Logistics resources space supply	Density of scale above logistics parks	no./kilometer ^2^	8.96%	*X*_*7*_
		Density of public logistics facilities	kilometer/kilometer^2^	11.59%	*X*_*8*_
Time matching	Logistics resources planned time	Logistics efficiency index	/	10.95%	*X*_*9*_

The TOPSIS method is used to calculate the match level of China’s nine logistics regions, which is shown in Table 4:

**Table 4:** Match-level measurement results.

Logistics regions	Year	Quantity matching index	Structure matching	Space matching	Time matching	Total matching
North China Logistics Region	2011	0.404	0.898	0.412	0.523	0.524
	2012	0.432	0.580	0.438	0.605	0.493
	2013	0.501	0.535	0.416	0.478	0.469
	2014	0.572	0.527	0.565	0.482	0.559
	2015	0.390	0.444	0.547	0.336	0.467
	2016	0.341	0.459	0.504	0.438	0.449
	2017	0.513	0.404	0.474	0.520	0.458
	2018	0.601	0.399	0.558	0.446	0.502
	2019	0.644	0.393	0.550	0.306	0.492
	2020	0.583	0.338	0.527	0.530	0.473
Northeast Logistics Region	2011	0.336	0.850	0.519	0.521	0.517
	2012	0.467	0.478	0.585	0.460	0.516
	2013	0.530	0.486	0.554	0.408	0.508
	2014	0.559	0.429	0.544	0.327	0.487
	2015	0.450	0.405	0.537	0.772	0.502
	2016	0.502	0.393	0.415	0.300	0.408
	2017	0.584	0.456	0.471	0.388	0.469
	2018	0.598	0.421	0.414	0.393	0.439
	2019	0.619	0.386	0.496	0.463	0.512
	2020	0.414	0.643	0.471	0.630	0.521
Shandong Peninsula Logistics Region	2011	0.526	0.761	0.521	0.449	0.569
	2012	0.514	0.686	0.480	0.483	0.547
	2013	0.462	0.511	0.380	0.153	0.423
	2014	0.337	0.664	0.485	0.327	0.469
	2015	0.376	0.530	0.486	0.288	0.441
	2016	0.436	0.471	0.545	0.523	0.491
	2017	0.509	0.492	0.552	0.627	0.516
	2018	0.580	0.353	0.560	0.625	0.504
	2019	0.611	0.157	0.543	0.795	0.488
	2020	0.644	0.381	0.447	0.394	0.434
the Yangtze River Delta Logistics region	2011	0.236	0.728	0.431	0.496	0.484
	2012	0.329	0.629	0.453	0.558	0.480
	2013	0.406	0.592	0.360	0.473	0.422
	2014	0.484	0.568	0.437	0.470	0.457
	2015	0.418	0.474	0.475	0.374	0.437
	2016	0.454	0.413	0.520	0.589	0.480
	2017	0.555	0.501	0.555	0.557	0.534
	2018	0.673	0.465	0.568	0.508	0.555
	2019	0.699	0.268	0.597	0.366	0.514
	2020	0.745	0.369	0.592	0.275	0.526
Southeast Coastal Logistics Region	2011	0.262	0.691	0.354	0.629	0.437
	2012	0.315	0.606	0.321	0.400	0.385
	2013	0.345	0.547	0.375	0.538	0.415
	2014	0.411	0.611	0.402	0.343	0.433
	2015	0.458	0.515	0.392	0.400	0.429
	2016	0.504	0.449	0.475	0.540	0.470
	2017	0.560	0.464	0.550	0.611	0.535
	2018	0.620	0.431	0.607	0.565	0.565
	2019	0.699	0.313	0.758	0.361	0.595
	2020	0.822	0.362	0.744	0.278	0.602
the Pearl River Delta Logistics Region	2011	0.229	0.375	0.349	0.416	0.335
	2012	0.294	0.310	0.334	0.463	0.333
	2013	0.347	0.639	0.408	0.524	0.456
	2014	0.476	0.526	0.430	0.368	0.452
	2015	0.455	0.471	0.429	0.428	0.429
	2016	0.525	0.486	0.511	0.292	0.465
	2017	0.617	0.531	0.567	0.503	0.549
	2018	0.667	0.515	0.604	0.502	0.572
	2019	0.733	0.508	0.677	0.563	0.623
	2020	0.632	0.584	0.682	0.605	0.645
Central Logistics Region	2011	0.248	0.574	0.481	0.429	0.471
	2012	0.382	0.586	0.445	0.686	0.496
	2013	0.401	0.547	0.461	0.519	0.471
	2014	0.436	0.557	0.512	0.383	0.487
	2015	0.430	0.480	0.476	0.239	0.434
	2016	0.512	0.470	0.491	0.494	0.482
	2017	0.618	0.483	0.520	0.662	0.526
	2018	0.721	0.449	0.503	0.546	0.519
	2019	0.613	0.465	0.513	0.289	0.485
	2020	0.633	0.408	0.572	0.418	0.512
Northwest Logistics Region	2011	0.296	0.658	0.495	0.642	0.509
	2012	0.380	0.433	0.535	0.545	0.484
	2013	0.409	0.537	0.533	0.502	0.498
	2014	0.553	0.519	0.456	0.424	0.480
	2015	0.422	0.592	0.403	0.380	0.428
	2016	0.424	0.496	0.474	0.330	0.445
	2017	0.571	0.500	0.466	0.358	0.470
	2018	0.679	0.413	0.425	0.362	0.468
	2019	0.643	0.469	0.588	0.371	0.529
	2020	0.651	0.404	0.570	0.751	0.556
Southwest Logistics Region	2011	0.220	0.714	0.451	0.475	0.456
	2012	0.293	0.551	0.457	0.443	0.427
	2013	0.402	0.537	0.470	0.511	0.457
	2014	0.462	0.483	0.472	0.523	0.465
	2015	0.476	0.487	0.458	0.487	0.477
	2016	0.532	0.477	0.498	0.571	0.522
	2017	0.633	0.534	0.475	0.537	0.526
	2018	0.691	0.445	0.526	0.597	0.557
	2019	0.634	0.356	0.599	0.306	0.510
	2020	0.658	0.417	0.574	0.242	0.504

### C. Evaluation results of the match level

#### 1) Solution for individualized characteristics

Utilizing Eq (13), the weights corresponding to the individualized characteristics are determined for each of the distinct logistics regions for every year under consideration. The outcomes of these calculations are systematically detailed in Tables 5–13.

**Table 5:** Weights of the individualized characteristics in the north China logistics region (2011–2020).

Index	X_1_	X_2_	X_3_	X_4_	X_5_	X_6_	X_7_	X_8_	X_9_
2011	0.0034	0.0284	**0.41**	**0.41**	**0.1062**	0.0034	0.0034	0.006	0.0293
2012	0.0077	0.0423	**0.1187**	0.0086	0.0463	0.008	0.0087	0.0166	**0.7431**
2013	0.0941	**0.1332**	**0.4448**	0.028	0.0662	0.0438	0.0519	0.0254	**0.1127**
2014	0.0374	0.0414	0.0173	0.0053	**0.4301**	0.0115	0.0103	**0.4301**	0.0166
2015	0.0529	0.0383	0.0593	0.0345	**0.2617**	**0.1569**	**0.1016**	**0.2626**	0.0321
2016	0.0555	0.0365	**0.1158**	0.0439	**0.1146**	0.0476	**0.1857**	**0.2977**	**0.1028**
2017	**0.1638**	**0.1045**	0.0542	0.0288	0.0273	0.0282	**0.2104**	**0.1671**	**0.2156**
2018	**0.1167**	**0.1151**	0.0118	0.0067	0.0097	**0.5583**	**0.121**	0.0425	0.0182
2019	**0.3107**	**0.3107**	0.0058	0.0026	0.004	0.0462	**0.3107**	0.0068	0.0026
2020	0.0649	0.0999	0.0054	0.0064	0.0054	**0.6509**	0.0993	0.0147	0.0532
Average	0.0907	0.0950	**0.1243**	0.0575	**0.1071**	**0.1555**	**0.1103**	**0.1270**	**0.1326**

**Table 6:** Weights of the individualized characteristics in the northeast logistics region (2011–2020).

Index	X_1_	X_2_	X_3_	X_4_	X_5_	X_6_	X_7_	X_8_	X_9_
2011	0.0026	0.0087	0.0214	**0.3159**	**0.3159**	0.0091	**0.3159**	0.0026	0.0079
2012	0.0166	**0.432**	0.0234	0.0085	**0.2575**	0.0216	**0.1228**	**0.1011**	0.0164
2013	0.0267	**0.5896**	0.0109	0.0094	0.0433	0.0216	0.0434	**0.2451**	0.0098
2014	0.0259	**0.4541**	0.0042	0.0057	0.0171	0.0103	0.0245	**0.4541**	0.0042
2015	0.0492	0.0042	0.0042	0.0058	0.0135	**0.334**	0.0251	0.0503	**0.5136**
2016	**0.7175**	0.0214	0.0229	0.0188	0.022	0.0174	0.0759	0.0866	0.0174
2017	**0.2103**	0.0333	0.0064	0.0056	0.0059	**0.4939**	0.0071	**0.2316**	0.0059
2018	**0.6059**	**0.1174**	0.0128	0.0111	0.0092	**0.106**	0.0132	**0.1109**	0.0136
2019	**0.5134**	**0.1073**	0.0059	0.0042	0.0403	**0.1114**	0.0049	**0.2036**	0.009
2020	0.0121	0.0128	**0.6341**	0.0081	0.0344	**0.1303**	0.0052	**0.1235**	0.0394
Average	**0.2180**	**0.1781**	0.0746	0.0393	0.0759	**0.1256**	0.0638	**0.1609**	0.0637

**Table 7:** Weights of the individualized characteristics in Shandong Peninsula Logistics Region (2011–2020).

Index	X_1_	X_2_	X_3_	X_4_	X_5_	X_6_	X_7_	X_8_	X_9_
2011	0.0016	**0.198**	**0.198**	**0.198**	**0.198**	0.0021	0.0016	**0.198**	0.0048
2012	0.0227	0.0581	**0.2242**	**0.2613**	**0.112**	0.0102	0.0152	**0.2604**	0.036
2013	0.0704	0.091	**0.4228**	0.0802	**0.1485**	0.0317	0.0465	0.0793	0.0296
2014	0.049	0.0128	0.0951	**0.5829**	0.041	0.0211	**0.1066**	0.0689	0.0225
2015	**0.1313**	0.0266	0.0851	**0.1887**	0.0576	0.0502	**0.2844**	**0.1382**	0.0379
2016	**0.1204**	0.0204	0.0389	0.0782	0.0309	0.0496	**0.3085**	**0.2845**	0.0686
2017	0.0913	0.0213	0.0247	0.0739	0.0177	0.0625	**0.4826**	**0.1282**	0.0978
2018	**0.1308**	0.0135	0.0075	0.0154	0.0076	**0.1168**	**0.6101**	0.0492	0.0492
2019	**0.165**	0.0073	0.0027	0.0027	0.0032	**0.3266**	**0.1487**	0.0171	**0.3266**
2020	0.0121	0.0128	**0.6341**	0.0081	0.0344	**0.1303**	0.0052	**0.1235**	0.0394
Average	**0.1104**	0.0458	**0.1102**	**0.1494**	0.0619	0.0992	**0.2326**	**0.1227**	0.0679

**Table 8:** Weights of the individualized characteristics in the Yangtze River Delta Logistics Region (2011–2020).

Index	X_1_	X_2_	X_3_	X_4_	X_5_	X_6_	X_7_	X_8_	X_9_
2011	0.0025	0.0025	**0.3083**	**0.3083**	**0.3083**	0.0025	0.0025	0.0452	0.0196
2012	0.0114	0.0171	0.0645	**0.3296**	**0.1087**	0.014	0.0148	**0.1426**	**0.2973**
2013	0.0457	0.0896	**0.317**	**0.2187**	0.0328	0.042	0.0592	0.03	**0.165**
2014	0.0115	0.0537	0.0281	0.0788	0.0063	0.0106	0.0162	**0.7615**	0.0334
2015	0.0701	0.0457	0.0566	**0.1696**	0.0508	0.062	0.0883	**0.406**	0.0509
2016	0.0202	0.0122	0.012	0.0186	0.0169	0.0267	0.0273	**0.1528**	**0.7132**
2017	0.0667	0.0616	0.0419	0.0645	0.0788	**0.1796**	0.0778	0.0702	**0.3589**
2018	0.0825	**0.1108**	0.0109	0.0247	**0.1132**	**0.5729**	0.0333	0.0072	0.0444
2019	**0.2717**	**0.158**	0.0096	0.0063	0.0399	**0.1469**	**0.1243**	**0.2319**	0.0115
2020	**0.3053**	**0.3053**	0.0025	0.0128	0.0377	0.0237	**0.3053**	0.0048	0.0025
Average	0.0887	0.0857	0.0851	**0.1232**	0.0793	**0.1081**	0.0749	**0.1852**	**0.1697**

**Table 9:** Weights of the individualized characteristics in Southeast Coastal Logistics Region (2011–2020).

Index	X_1_	X_2_	X_3_	X_4_	X_5_	X_6_	X_7_	X_8_	X_9_
2011	0.003	0.003	**0.3652**	**0.2473**	0.003	0.0046	0.0033	0.0053	**0.3652**
2012	0.0255	0.0245	**0.1936**	**0.6181**	0.0224	0.0209	0.0209	0.0294	0.0446
2013	0.0502	0.0421	**0.2317**	**0.1612**	0.0421	0.0364	0.0747	0.0417	**0.3197**
2014	0.014	0.0113	0.0472	**0.8719**	0.0128	0.0088	0.0146	0.0089	0.0104
2015	0.0936	0.0658	**0.1161**	**0.4229**	0.0763	0.0482	0.0626	0.0366	0.078
2016	0.0952	0.056	0.0649	**0.1102**	0.0609	0.0412	**0.2841**	0.0268	**0.2606**
2017	0.0565	0.0324	0.0262	0.0815	0.0443	0.0209	**0.1042**	0.0259	**0.6081**
2018	**0.1744**	0.0604	0.0445	0.0459	0.0948	**0.1636**	0.095	0.0524	**0.2691**
2019	**0.1073**	0.0189	0.0055	0.0032	0.0417	0.0422	**0.388**	**0.388**	0.0052
2020	**0.2129**	**0.2129**	0.0018	0.0108	**0.2129**	**0.2129**	**0.1251**	0.0091	0.0018
Average	0.0833	0.0527	**0.1097**	**0.2573**	0.0611	0.0600	**0.1173**	0.0624	**0.1963**

**Table 10:** Weights of the individualized characteristics in the Pearl River Delta Logistics Region (2011–2020).

Index	X_1_	X_2_	X_3_	X_4_	X_5_	X_6_	X_7_	X_8_	X_9_
2011	0.0852	0.0852	0.0852	**0.1535**	**0.1192**	0.0852	0.0888	0.0901	**0.2075**
2012	0.0976	0.0961	0.0925	0.0878	0.0775	0.0888	0.0775	0.0787	**0.3035**
2013	0.0102	0.0115	0.0118	**0.8495**	0.02	0.0092	0.0151	0.007	0.0658
2014	0.0702	**0.2734**	**0.1048**	**0.1166**	**0.1755**	0.0623	0.0845	0.0449	0.068
2015	0.088	**0.1798**	**0.1588**	0.0762	0.0656	**0.1722**	0.0772	0.053	**0.1293**
2016	0.06	**0.2403**	**0.1274**	0.0421	0.0486	**0.1468**	**0.2649**	0.0424	0.0274
2017	0.0246	**0.6479**	0.0845	0.0132	0.0242	0.0814	0.0345	0.0342	0.0556
2018	0.0535	**0.3679**	**0.18**	0.0104	0.0185	**0.1777**	0.0588	0.0778	0.0553
2019	0.0391	**0.2385**	**0.2385**	0.002	0.0073	0.0902	**0.2385**	**0.1017**	0.0441
2020	**0.1714**	0.0051	**0.1317**	0.0023	**0.1714**	**0.1714**	0.0038	**0.1714**	**0.1714**
Average	0.0700	**0.2146**	**0.1215**	**0.1354**	0.0728	**0.1085**	0.0944	0.0701	**0.1128**

**Table 11:** Weights of individualized characteristics in Central Logistics Region (2011–2020).

Index	X_1_	X_2_	X_3_	X_4_	X_5_	X_6_	X_7_	X_8_	X_9_
2011	0.0033	0.0033	**0.4032**	0.0033	**0.1661**	0.0053	0.0033	**0.4032**	0.0089
2012	0.0071	0.0145	0.0798	0.0223	**0.1478**	0.0069	0.0072	0.0104	**0.7041**
2013	0.0486	0.0665	**0.204**	**0.124**	**0.1692**	**0.121**	0.0485	0.049	**0.1692**
2014	0.0065	0.01	0.0195	0.0332	**0.4553**	**0.4553**	0.0074	0.0053	0.0075
2015	0.0735	0.0806	**0.1089**	**0.163**	**0.21**	**0.1951**	0.0903	0.0411	0.0375
2016	0.0915	**0.1072**	0.0823	**0.1329**	**0.1983**	0.0978	**0.1247**	0.0312	**0.1341**
2017	0.0492	**0.1129**	0.0276	0.0705	0.0202	0.0717	0.0712	0.0279	**0.5489**
2018	0.054	**0.7554**	0.0185	0.012	0.0086	0.0187	0.081	0.0074	0.0443
2019	**0.4127**	0.0351	0.0297	0.0582	0.0123	0.0123	**0.3887**	0.036	0.0152
2020	**0.3158**	0.0077	0.0026	**0.3158**	0.0046	0.0196	**0.3158**	0.0118	0.0064
Average	**0.1062**	**0.1193**	0.0976	0.0935	**0.1392**	**0.1004**	**0.1138**	0.0623	**0.1676**

**Table 12:** Weights of the individualized characteristics in northwest Logistics Region (2011–2020).

Index	X_1_	X_2_	X_3_	X_4_	X_5_	X_6_	X_7_	X_8_	X_9_
2011	0.0038	0.0038	**0.4584**	0.0223	**0.4584**	0.0038	0.0104	0.0038	0.0353
2012	0.0265	0.0552	**0.2625**	0.0235	**0.3031**	**0.1373**	0.0548	0.0557	0.0814
2013	0.0159	0.008	0.0349	0.0272	0.0161	0.0727	0.0136	**0.7952**	0.0166
2014	0.0901	**0.1132**	0.059	**0.1601**	0.0359	0.0309	0.0353	**0.439**	0.0365
2015	0.0172	0.011	0.0149	**0.8953**	0.0074	0.0258	0.0074	0.0117	0.0093
2016	0.0816	0.0626	0.0673	**0.3167**	0.0943	**0.1555**	0.0517	**0.1325**	0.0378
2017	0.0683	**0.1195**	0.0199	**0.5517**	0.0346	**0.1143**	0.0215	0.0504	0.0197
2018	0.0502	**0.8446**	0.0105	0.0245	0.0102	0.0109	0.0074	0.0337	0.008
2019	0.0736	0.0175	0.0049	0.0287	0.0063	**0.4164**	**0.4164**	0.0322	0.0041
2020	**0.3432**	0.0102	0.0028	0.0161	0.0054	**0.1995**	0.0545	0.0251	**0.3432**
Average	0.0770	**0.1246**	0.0935	**0.2066**	0.0972	**0.1167**	0.0673	**0.1579**	0.0592

**Table 13:** Weights of the individualized characteristics in Southwest Logistics Region (2011–2020).

Index	X_1_	X_2_	X_3_	X_4_	X_5_	X_6_	X_7_	X_8_	X_9_
2011	0.0035	0.0035	**0.4216**	**0.3686**	0.005	**0.1522**	0.0035	0.0205	0.0216
2012	0.0091	0.0088	0.0713	0.014	0.0067	**0.8128**	0.0084	0.0402	0.0287
2013	0.0125	0.0187	0.0286	0.0321	0.0068	0.0213	0.0094	**0.8022**	0.0683
2014	0.0551	0.092	0.0589	0.0864	0.0357	0.0649	0.0334	**0.2898**	**0.2838**
2015	0.0979	0.0929	0.0641	**0.195**	**0.1515**	0.0289	0.041	**0.1226**	**0.2061**
2016	0.033	0.0279	0.0143	0.0311	**0.6013**	0.0219	0.0107	0.008	**0.2518**
2017	0.0458	**0.1187**	0.0114	**0.6364**	0.0454	0.0395	0.0088	0.0058	0.0882
2018	0.0396	**0.3005**	0.0044	0.0116	**0.3005**	0.0349	0.0053	0.0028	**0.3005**
2019	**0.4118**	0.0965	0.0229	0.0139	**0.1063**	**0.1373**	**0.152**	0.0426	0.0168
2020	**0.3756**	0.0207	0.0031	0.081	0.0147	**0.1231**	**0.3756**	0.0031	0.0031
Average	**0.1084**	0.0780	0.0701	**0.1470**	**0.1274**	**0.1437**	0.0648	**0.1338**	**0.1269**

#### 2) Evaluation of the match level based on individualized characteristics

In accordance with Eq (14), the assessment outcomes for each logistics region within the specified period from 2011 to 2020 are derived and subsequently organized in Tables 14–22.

**Table 14:** Evaluation results for the north China Logistics Region (2011–2020).

Year	2011	2012	2013	2014	2015	2016	2017	2018	2019	2020
2011	0.064	0.280	0.152	0.368	0.309	0.333	0.338	0.644	0.500	0.729
2012	0.398	0.086	0.173	0.338	0.279	0.282	0.284	0.565	0.441	0.640
2013	0.450	0.392	0.175	0.558	0.377	0.378	0.293	0.487	0.336	0.555
2014	0.423	0.383	0.241	0.066	0.132	0.156	0.192	0.354	0.249	0.406
2015	0.499	0.748	0.377	0.219	0.179	0.215	0.302	0.297	0.412	0.321
2016	0.489	0.496	0.338	0.320	0.263	0.210	0.269	0.566	0.468	0.643
2017	0.544	0.304	0.364	0.500	0.354	0.228	0.178	0.598	0.254	0.692
2018	0.544	0.482	0.366	0.414	0.261	0.205	0.178	0.085	0.134	0.091
2019	0.551	0.823	0.357	0.479	0.302	0.271	0.270	0.151	0.056	0.180
2020	0.622	0.296	0.500	0.554	0.347	0.278	0.172	0.087	0.150	0.081

**Table 15:** Evaluation results for the northeast Logistics Region (2011–2020).

Year	2011	2012	2013	2014	2015	2016	2017	2018	2019	2020
2011	0.056	0.286	0.448	0.571	0.387	0.795	0.451	0.684	0.615	0.291
2012	0.352	0.098	0.120	0.157	0.451	0.545	0.371	0.466	0.401	0.417
2013	0.338	0.133	0.087	0.097	0.490	0.382	0.314	0.328	0.282	0.532
2014	0.355	0.153	0.075	0.067	0.580	0.305	0.340	0.265	0.230	0.670
2015	0.383	0.506	0.654	0.521	0.072	0.240	0.124	0.238	0.216	0.699
2016	0.485	0.505	0.600	0.504	0.675	0.145	0.557	0.204	0.214	0.630
2017	0.491	0.308	0.237	0.189	0.471	0.133	0.070	0.108	0.102	0.561
2018	0.552	0.335	0.208	0.203	0.478	0.129	0.179	0.105	0.116	0.596
2019	0.488	0.185	0.144	0.126	0.395	0.112	0.115	0.076	0.072	0.592
2020	0.467	0.352	0.423	0.338	0.224	0.527	0.196	0.448	0.383	0.080

**Table 16:** Evaluation results for the Shandong Peninsula Logistics Region (2011–2020).

Year	2011	2012	2013	2014	2015	2016	2017	2018	2019	2020
2011	0.045	0.060	0.110	0.144	0.350	0.371	0.547	0.696	0.452	0.590
2012	0.136	0.111	0.144	0.168	0.287	0.307	0.451	0.575	0.446	0.516
2013	0.252	0.268	0.189	0.408	0.319	0.359	0.458	0.561	0.530	0.499
2014	0.280	0.188	0.229	0.124	0.169	0.207	0.225	0.268	0.404	0.352
2015	0.306	0.235	0.299	0.249	0.173	0.197	0.205	0.233	0.397	0.311
2016	0.302	0.238	0.333	0.298	0.158	0.137	0.151	0.178	0.275	0.249
2017	0.287	0.236	0.340	0.256	0.141	0.128	0.113	0.126	0.200	0.196
2018	0.329	0.307	0.427	0.392	0.171	0.135	0.099	0.081	0.147	0.110
2019	0.400	0.413	0.502	0.652	0.248	0.168	0.128	0.098	0.057	0.075
2020	0.402	0.413	0.480	0.328	0.215	0.326	0.173	0.092	0.241	0.057

**Table 17:** Evaluation results for the Yangtze River Delta Logistics Region (2011–2020).

Year	2011	2012	2013	2014	2015	2016	2017	2018	2019	2020
2011	0.056	0.133	0.156	0.209	0.186	0.290	0.278	0.650	0.410	0.582
2012	0.175	0.108	0.181	0.224	0.176	0.153	0.213	0.539	0.356	0.482
2013	0.367	0.276	0.191	0.839	0.470	0.377	0.281	0.557	0.405	0.414
2014	0.387	0.216	0.212	0.087	0.138	0.343	0.260	0.509	0.279	0.348
2015	0.359	0.293	0.301	0.222	0.177	0.562	0.329	0.432	0.268	0.374
2016	0.365	0.226	0.293	0.178	0.167	0.084	0.144	0.321	0.232	0.335
2017	0.270	0.190	0.225	0.351	0.214	0.161	0.121	0.184	0.195	0.246
2018	0.280	0.236	0.263	0.681	0.377	0.291	0.157	0.076	0.229	0.166
2019	0.456	0.442	0.395	0.167	0.214	0.579	0.307	0.149	0.087	0.115
2020	0.384	0.390	0.409	0.611	0.346	0.794	0.409	0.215	0.196	0.055

**Table 18:** Evaluation results for the Southeast Coastal Logistics Region (2011–2020).

Year	2011	2012	2013	2014	2015	2016	2017	2018	2019	2020
2011	0.060	0.098	0.133	0.110	0.181	0.334	0.155	0.293	0.537	0.467
2012	0.309	0.159	0.286	0.180	0.202	0.395	0.480	0.362	0.572	0.465
2013	0.238	0.329	0.203	0.443	0.269	0.272	0.250	0.293	0.496	0.432
2014	0.370	0.123	0.326	0.093	0.168	0.345	0.567	0.351	0.497	0.385
2015	0.364	0.241	0.303	0.284	0.210	0.331	0.472	0.313	0.544	0.362
2016	0.318	0.366	0.232	0.475	0.270	0.180	0.231	0.231	0.454	0.322
2017	0.291	0.295	0.200	0.363	0.216	0.152	0.118	0.189	0.326	0.279
2018	0.320	0.443	0.222	0.595	0.309	0.186	0.191	0.146	0.311	0.203
2019	0.517	0.700	0.383	0.960	0.482	0.266	0.535	0.252	0.062	0.140
2020	0.579	0.351	0.441	0.390	0.244	0.302	0.671	0.303	0.195	0.046

**Table 19:** Evaluation results for the Pearl River Delta Logistics Region (2011–2020).

Year	2011	2012	2013	2014	2015	2016	2017	2018	2019	2020
2011	0.321	0.336	0.699	0.404	0.375	0.455	0.728	0.507	0.478	0.407
2012	0.323	0.306	0.879	0.396	0.351	0.437	0.655	0.463	0.453	0.396
2013	0.223	0.232	0.092	0.302	0.295	0.347	0.570	0.406	0.370	0.341
2014	0.275	0.320	0.560	0.224	0.265	0.290	0.301	0.281	0.303	0.340
2015	0.275	0.279	0.738	0.281	0.239	0.302	0.378	0.279	0.311	0.325
2016	0.310	0.371	0.757	0.238	0.229	0.182	0.259	0.212	0.207	0.325
2017	0.203	0.186	0.742	0.178	0.149	0.175	0.101	0.121	0.171	0.210
2018	0.208	0.180	0.837	0.190	0.134	0.141	0.118	0.101	0.131	0.183
2019	0.191	0.132	0.935	0.168	0.106	0.072	0.071	0.057	0.049	0.121
2020	0.157	0.114	0.735	0.198	0.137	0.231	0.376	0.219	0.214	0.041

**Table 20:** Evaluation results for the Central Logistics Region (2011–2020).

Year	2011	2012	2013	2014	2015	2016	2017	2018	2019	2020
2011	0.064	0.477	0.235	0.407	0.296	0.284	0.414	0.839	0.628	0.602
2012	0.355	0.084	0.183	0.472	0.261	0.219	0.155	0.536	0.565	0.478
2013	0.402	0.342	0.194	0.334	0.219	0.220	0.290	0.576	0.503	0.431
2014	0.421	0.549	0.192	0.068	0.146	0.192	0.441	0.518	0.463	0.381
2015	0.504	0.780	0.271	0.306	0.213	0.247	0.616	0.574	0.430	0.374
2016	0.526	0.383	0.221	0.346	0.213	0.194	0.311	0.453	0.346	0.316
2017	0.411	0.170	0.216	0.412	0.223	0.201	0.114	0.260	0.272	0.250
2018	0.507	0.327	0.256	0.516	0.281	0.243	0.243	0.087	0.205	0.294
2019	0.427	0.717	0.329	0.709	0.343	0.301	0.555	0.494	0.122	0.183
2020	0.509	0.516	0.299	0.421	0.235	0.226	0.394	0.487	0.074	0.056

**Table 21:** Evaluation results for the northwest Logistics Region (2011–2020).

Year	2011	2012	2013	2014	2015	2016	2017	2018	2019	2020
2011	0.068	0.187	0.879	0.516	0.408	0.292	0.324	0.932	0.542	0.456
2012	0.209	0.169	0.570	0.383	0.985	0.384	0.621	0.609	0.365	0.422
2013	0.391	0.268	0.089	0.171	0.485	0.217	0.329	0.845	0.351	0.349
2014	0.516	0.362	0.216	0.168	0.377	0.237	0.269	0.423	0.546	0.408
2015	0.618	0.418	0.699	0.412	0.095	0.218	0.162	0.764	0.523	0.439
2016	0.497	0.334	0.471	0.300	0.342	0.214	0.252	0.724	0.457	0.473
2017	0.594	0.380	0.518	0.306	0.177	0.181	0.145	0.357	0.452	0.416
2018	0.587	0.397	0.405	0.257	0.526	0.273	0.345	0.092	0.579	0.422
2019	0.564	0.356	0.289	0.193	0.342	0.172	0.223	0.413	0.065	0.355
2020	0.623	0.379	0.298	0.204	0.414	0.192	0.268	0.491	0.120	0.059

**Table 22:** Evaluation results for the Southwest Logistics Region (2011–2020).

Year	2011	2012	2013	2014	2015	2016	2017	2018	2019	2020
2011	0.065	0.138	0.366	0.223	0.236	0.568	0.166	0.454	0.505	0.585
2012	0.316	0.090	0.364	0.240	0.289	0.677	0.505	0.469	0.443	0.517
2013	0.304	0.500	0.090	0.151	0.233	0.658	0.336	0.396	0.391	0.467
2014	0.366	0.527	0.248	0.164	0.213	0.530	0.367	0.324	0.348	0.438
2015	0.387	0.896	0.431	0.227	0.187	0.309	0.287	0.269	0.332	0.436
2016	0.369	0.482	0.783	0.302	0.183	0.087	0.323	0.171	0.258	0.373
2017	0.324	0.334	0.843	0.321	0.169	0.236	0.080	0.156	0.218	0.352
2018	0.400	0.249	0.829	0.310	0.177	0.070	0.326	0.055	0.174	0.306
2019	0.546	0.293	0.510	0.357	0.321	0.349	0.708	0.347	0.130	0.177
2020	0.472	0.171	0.886	0.453	0.290	0.412	0.178	0.386	0.101	0.061

The assessment outcomes for each column are systematically arranged from lowest to highest to establish the ranking for the logistics architecture and resource alignment evaluation, tailored to individual characteristics, for each logistics region within various years. These rankings are comprehensively presented in Tables 23–31.

**Table 23:** Evaluation ranking of north China Logistics Region on individualized characteristics (2011–2020).

Year	2011	2012	2013	2014	2015	2016	2017	2018	2019	2020
2011	1	2	4	3	7	8	2	9	10	10
2012	3	1	3	5	8	10	9	10	9	9
2013	2	8	1	1	6	9	7	8	8	8
2014	5	9	2	2	5	7	8	5	7	7
2015	7	10	5	4	4	4	4	4	6	6
2016	6	7	7	6	3	2	5	3	5	5
2017	4	6	9	8	1	3	1	2	4	4
2018*	8	4	8	7	2	1	6	1	3	3
2019▽	10	5	6	9	10	5	10	6	2	2
2020	9	3	10	10	9	6	3	7	1	1

Note: Rankings should be based on the perspective that is most beneficial to the evaluated object, where

* indicates individualized characteristics and

▽ indicates a lack of individualized characteristics.

**Table 24:** Evaluation ranking of northeast Logistics Region based on individualized characteristics (2011–2020).

Year	2011	2012	2013	2014	2015	2016	2017	2018	2019	2020
2011▽	1	5	8	10	3	10	9	10	10	2
2012	3	1	3	4	5	9	8	9	9	3
2013	2	2	2	2	8	7	6	7	7	4
2014	4	3	1	1	9	6	7	6	6	9
2015	5	10	10	9	1	5	3	5	5	10
2016	7	9	9	8	10	4	10	4	4	8
2017	9	6	6	5	6	3	1	3	2	5
2018	10	7	5	6	7	2	4	2	3	7
2019*	8	4	4	3	4	1	2	1	1	6
2020	6	8	7	7	2	8	5	8	8	1

Note: Rankings should be based on the perspective that is most beneficial to the evaluated object, where

* indicates individualized characteristics and

▽ indicates a lack of individualized characteristics.

**Table 25:** Evaluation ranking of Shandong Peninsula Logistics Region based on individualized characteristics (2011–2020).

Year	2011	2012	2013	2014	2015	2016	2017	2018	2019	2020
2011▽	1	1	2	2	10	10	10	10	9	10
2012	2	2	1	3	8	7	8	9	8	9
2013	3	7	3	9	9	9	9	8	10	8
2014	4	3	4	1	3	6	7	7	7	7
2015	7	4	7	4	5	5	6	6	6	6
2016	6	6	6	6	2	3	4	5	5	5
2017	5	5	5	5	1	1	2	4	3	4
2018*	8	8	8	8	4	2	1	1	2	3
2019	9	9	10	10	7	4	3	3	1	2
2020	10	10	9	7	6	8	5	2	4	1

Note: Rankings should be based on the perspective that is most beneficial to the evaluated object, where

* indicates individualized characteristics and

▽ indicates a lack of individualized characteristics.

**Table 26:** Evaluation ranking of the Yangtze River Delta Logistics Region based on individualized characteristics (2011–2020).

Year	2011	2012	2013	2014	2015	2016	2017	2018	2019	2020
2011*	1	2	1	4	5	4	6	10	10	10
2012	2	1	2	6	3	2	4	8	8	9
2013	7	7	3	10	10	7	7	9	9	8
2014	9	4	4	1	1	6	5	7	7	6
2015	5	8	8	5	4	8	9	6	6	7
2016	6	5	7	3	2	1	2	5	5	5
2017	3	3	5	7	6	3	1	3	2	4
2018	4	6	6	9	9	5	3	1	4	3
2019	10	10	9	2	7	9	8	2	1	2
2020▽	8	9	10	8	8	10	10	4	3	1

Note: Rankings should be based on the perspective that is most beneficial to the evaluated object, where

* indicates individualized characteristics and

▽ indicates a lack of individualized characteristics.

**Table 27:** Evaluation ranking of Southeast Coastal Logistics Region based on individualized characteristics (2011–2020).

Year	2011	2012	2013	2014	2015	2016	2017	2018	2019	2020
2011*	1	1	1	2	8	2	6	8	8	10
2012▽	4	3	6	3	10	7	10	10	10	9
2013	2	6	3	7	5	5	5	6	6	8
2014	8	2	8	1	9	9	9	7	7	7
2015	7	4	7	4	7	6	8	9	9	6
2016	5	8	5	8	2	4	3	5	5	5
2017	3	5	2	5	1	1	2	4	4	4
2018	6	9	4	9	3	3	1	3	3	3
2019	9	10	9	10	4	8	4	1	1	2
2020	10	7	10	6	6	10	7	2	2	1

Note: Rankings should be based on the perspective that is most beneficial to the evaluated object, where

* indicates individualized characteristics and

▽ indicates a lack of individualized characteristics.

**Table 28:** Evaluation ranking of the Pearl River Delta Logistics Region based on individualized characteristics (2011–2020).

Year	2011	2012	2013	2014	2015	2016	2017	2018	2019	2020
2011▽	9	9	3	10	10	10	10	10	10	10
2012	10	7	9	9	9	9	9	9	9	9
2013	5	5	1	8	8	8	8	8	8	8
2014	6	8	2	5	7	6	5	7	6	7
2015	7	6	5	7	6	7	7	6	7	5
2016	8	10	7	6	5	4	4	4	4	6
2017	3	4	6	2	4	3	2	3	3	4
2018	4	3	8	3	2	2	3	2	2	3
2019*	2	2	10	1	1	1	1	1	1	2
2020	1	1	4	4	3	5	6	5	5	1

Note: Rankings should be based on the perspective that is most beneficial to the evaluated object, where

* indicates individualized characteristics and

▽ indicates a lack of individualized characteristics.

**Table 29:** Evaluation ranking of Central Logistics Region based on individualized characteristics (2011–2020).

Year	2011	2012	2013	2014	2015	2016	2017	2018	2019	2020
2011	1	6	6	5	9	9	7	10	10	10
2012	2	1	1	8	7	4	2	7	9	9
2013	3	4	3	3	4	5	4	9	8	8
2014*	5	8	2	1	1	1	8	6	7	7
2015	7	10	8	2	2	8	10	8	6	6
2016	10	5	5	4	3	2	5	3	5	5
2017	4	2	4	6	5	3	1	2	4	3
2018	8	3	7	9	8	7	3	1	3	4
2019▽	6	9	10	10	10	10	9	5	2	2
2020	9	7	9	7	6	6	6	4	1	1

Note: Rankings should be based on the perspective that is most beneficial to the evaluated object, where

* indicates individualized characteristics and

▽ indicates a lack of individualized characteristics.

**Table 30:** Evaluation ranking of northwest Logistics Region based on individualized characteristics (2011–2020).

Year	2011	2012	2013	2014	2015	2016	2017	2018	2019	2020
2011	1	2	10	10	6	9	7	10	8	9
2012▽	2	1	8	8	10	10	10	6	4	6
2013	3	3	1	2	8	5	8	9	3	2
2014	5	10	9	9	1	6	2	8	7	8
2015*	9	5	3	3	4	1	3	3	1	3
2016	4	6	2	1	5	7	6	4	9	4
2017	8	9	5	5	9	8	9	1	10	7
2018	7	8	7	7	2	2	1	2	5	5
2019	6	4	6	6	3	4	4	7	6	10
2020	10	7	4	4	7	3	5	5	2	1

Note: Rankings should be based on the perspective that is most beneficial to the evaluated object, where

* indicates individualized characteristics and

▽ indicates a lack of individualized characteristics.

**Table 31:** Evaluation ranking of Southwest Logistics Region based on individualized characteristics (2011–2020).

Year	2011	2012	2013	2014	2015	2016	2017	2018	2019	2020
2011	1	2	4	3	7	8	2	9	10	10
2012	3	1	3	5	8	10	9	10	9	9
2013	2	8	1	1	6	9	7	8	8	8
2014	5	9	2	2	5	7	8	5	7	7
2015	7	10	5	4	4	4	4	4	6	6
2016	6	7	7	6	3	2	5	3	5	5
2017	4	6	9	8	1	3	1	2	4	4
2018*	8	4	8	7	2	1	6	1	3	3
2019▽	10	5	6	9	10	5	10	6	2	2
2020	9	3	10	10	9	6	3	7	1	1

Note: Rankings should be based on the perspective that is most beneficial to the evaluated object, where

* indicates individualized characteristics and

▽ indicates a lack of individualized characteristics.

#### 3) Democratic match evaluation based on individualized characteristics

According to Eq (15), the democratic evaluation results of each logistics region for each year from 2011 to 2020 are calculated, as shown in Tables 32–40.

**Table 32:** Democratic evaluation values and rankings of the north China Logistics Region, 2011–2020.

Rank	Year	Evaluation value
1	2020	4.2
2	2014	4.5
3	2015	4.7
4	2011	5.3
5	2017	5.3
6	2012	5.6
7	2018	5.8
8	2016	6.1
9	2019	6.2
10	2013	7.3

**Table 33:** Democratic evaluation values and rankings of the northeast China Logistics Region, 2011–2020.

Rank	Year	Evaluation value
1	2019	3.9
2	2020	3.9
3	2013	4.7
4	2012	5.5
5	2015	5.5
6	2011	6
7	2014	6.2
8	2016	6.3
9	2017	6.3
10	2018	6.7

**Table 34:** Democratic evaluation values and rankings of the Shandong Peninsula Logistics Region, 2011–2020.

Rank	Year	Evaluation value
1	2011	5.6
2	2012	5.5
3	2013	5.5
4	2014	6.5
5	2015	6.4
6	2016	5.8
7	2017	5.2
8	2018	5.4
9	2019	4.6
10	2020	4.5

**Table 35:** Democratic evaluation values and rankings of the Yangtze River Delta Logistics Region, 2011–2020.

Rank	Year	Evaluation value
1	2011	5.2
2	2012	5.3
3	2013	6.5
4	2014	5
5	2015	5.4
6	2016	5
7	2017	4.7
8	2018	6.1
9	2019	6.1
10	2020	5.7

**Table 36:** Democratic evaluation values and rankings of the Southeastern Coastal Logistics Region, 2011–2020.

Rank	Year	Evaluation value
1	2018	4.5
2	2012	4.9
3	2017	5.1
4	2011	5.2
5	2013	5.4
6	2015	5.4
7	2016	5.7
8	2019	6.1
9	2014	6.3
10	2020	6.4

**Table 37:** Democratic evaluation values and rankings of the Pearl River Delta Logistics Region, 2011–2020.

Rank	Year	Evaluation value
1	2019	1.8
2	2020	2.4
3	2018	3.9
4	2017	4.5
5	2016	5.6
6	2015	6
7	2013	7.1
8	2014	7.3
9	2012	7.7
10	2011	8.7

**Table 38:** Democratic evaluation values and rankings of the Central Logistics Region, 2011–2020.

Rank	Year	Evaluation value
1	2018	4.7
2	2011	5.2
3	2015	5.4
4	2017	5.4
5	2019	5.5
6	2020	5.5
7	2012	5.6
8	2014	5.6
9	2013	5.9
10	2016	6.2

**Table 39:** Democratic evaluation values and rankings of the northwest Logistics Region, 2011–2020.

Rank	Year	Evaluation value
1	2020	3.8
2	2018	4.3
3	2016	4.5
4	2019	4.8
5	2012	4.9
6	2011	5.8
7	2014	6.3
8	2017	6.6
9	2013	7
10	2015年	7

**Table 40:** Democratic evaluation values and rankings of the Southwest Logistics Region, 2011–2020.

Rank	Year	Evaluation value
1	2018	4.2
2	2019	5
3	2017	5.1
4	2012	5.2
5	2014	5.5
6	2015	5.5
7	2020	5.8
8	2011	5.9
9	2013	6
10	2016	6.8

### D. Analysis of the evaluation results

#### 1) Identification of individualized characteristics

*North China logistics region*. On average, North China logistics region showed a lack of distinctive characteristics for indicators *X*_*1*_, *X*_*2*_, and *X*_*4*_, while other indicators maintained a relatively balanced performance. In the years 2016 and 2017, the region had five indicators that stood out for their individualized characteristics: *X*_*3*_, *X*_*5*_, *X*_*7*_, *X*_*8*_, and *X*_*9*_. In addition, the indicators *X*_*9*_ in 2012, *X*_*6*_ in 2018 and 2020 all exhibited obvious individualized characteristics, with advantage indicator weights exceeding 0.5.

*Northeast logistics region*. On average, Northeast logistics region demonstrated clear individualized characteristics for the indicators *X*_*1*_, *X*_*2*_, *X*_*6*_, and *X*_*7*_, while maintaining a balanced performance for these indicators. Notably, indicator *X*_*4*_ lagged significantly behind the others in performance. In 2012, 2018, and 2019, the region had four prominent indicators with individualized characteristics, namely, *X*_*2*_, *X*_*5*_, *X*_*7*_, *X*_*8*_; *X*_*1*_, *X*_*2*_, *X*_*6*_, *X*_*8*_; and *X*_*1*_, X_2_, X_6_, X_8_. Furthermore, in isolated instances, certain indicators showed a strong individualized presence with substantial weight factors over 0.5: *X*_*2*_ in 2013, *X*_*9*_ in 2015, and *X*_*1*_ in the years 2016, 2018, and 2019.

*Shandong peninsula logistics region*. On average, Shandong peninsula logistics region has displayed unique individualized characteristics for the indicators *X*_*1*_, *X*_*3*_, *X*_*4*_, *X*_*7*_, and *X*_*8*_, while maintaining a relatively balanced performance in these areas. Nonetheless, a considerable gap was observed between the performance of indicator *X*_*2*_ and that of the other indicators. In the year 2011, the region presented five indicators with marked individualized characteristics, which indicates a balanced development in the logistics matching for the Shandong Peninsula. Furthermore, in 2014, indicator *X*_*4*_, and in 2020, indicator *X*_*3*_, both displayed significant individualized features, with their corresponding advantage indicator weights exceeding 0.5.

*Yangtze river delta logistics region*. Yangtze river delta logistics region, on average, has shown distinct individualized characteristics for the indicators *X*_*4*_, *X*_*6*_, *X*_*8*_, and *X*_*9*_, maintaining a balanced performance among the nine indicators with only slight differences noted. In the year 2015, the region presented five indicators with marked individualized characteristics, which indicates a balanced development in logistics matching within the Yangtze River Delta. Additionally, in 2014, indicator *X*_*8*_, in 2016, indicator *X*_*9*_, and in 2018, indicator *X*_*6*_ each displayed significant individualized features, with their corresponding advantage indicator weights exceeding the critical value of 0.5.

*Southeast coastal logistics region*. Southeast coastal logistics region, on average, has shown distinct individualized characteristics for the indicators *X*_*3*_, *X*_*4*_, *X*_*7*_, and *X*_*9*_, while maintaining a balanced performance among the nine indicators with negligible differences observed. In 2020, the region presented five indicators with marked individualized characteristics, indicating a balanced development in logistics matching for the Southeast coastal logistics region. Furthermore, in the years 2012 and 2014, the *X*_*4*_ indicator displayed significant individualized features, with the corresponding advantage indicator weight exceeding the critical value of 0.5.

*Pearl river delta logistics region*. Pearl river delta logistics region, on average, has shown distinct individualized characteristics for indicators *X*_*2*_, *X*_*3*_, *X*_*4*_, *X*_*6*_, and *X*_*9*_, while maintaining a balanced performance among these indicators with only minor differences noted. In 2020, the region presented six indicators with marked individualized characteristics, which indicates a balanced development in logistics matching within the Pearl River Delta. Furthermore, in 2013, the *X*_*4*_ indicator, and in 2017, the *X*_*2*_ indicator, each demonstrated significant individualized features, with their corresponding advantage indicator weights exceeding the critical value of 0.5.

*Central logistics region*. Central logistics region, on average, has exhibited unique individualized characteristics for the indicators *X*_*1*_, *X*_*2*_, *X*_*5*_, *X*_*6*_, *X*_*7*_, and *X*_*9*_, while maintaining a balanced performance among these indicators with negligible differences observed. In 2013, the region presented five indicators with marked individualized characteristics, indicating a balanced development in logistics matching within the central logistics region. Furthermore, in the year 2012, indicator *X*_*9*_, in 2017, indicator *X*_*9*_, and in 2018, indicator *X*_*2*_, each displayed significant individualized features, with their corresponding advantage indicator weights exceeding the critical value of 0.5.

*Northwest logistics region*. Northwest logistics region, on average, has shown distinct individualized characteristics for indicators *X*_*2*_, *X*_*4*_, *X*_*6*_, and *X*_*8*_, while maintaining a balanced performance among the nine indicators. Across half of the years under review, namely 2012, 2014, 2016, 2017, and 2020, three indicators stood out with pronounced individualized characteristics. Furthermore, in 2013 for indicator *X*_*8*_, in 2015 for indicator *X*_*4*_, in 2017 for indicator *X*_*4*_, and in 2018 for indicator *X*_*2*_, there was a marked display of individualized features, with the advantage indicator weights in each case exceeding the critical value of 0.5.

*Southwest logistics region*. Southwest logistics region, on average, has exhibited distinct individualized characteristics for indicators *X*_*1*_, *X*_*4*_, *X*_*5*_, *X*_*6*_, *X*_*8*_, and *X*_*9*_, while maintaining a balanced performance among the nine indicators with only minor differences observed. During the years 2015 and 2019, four indicators with marked individualized characteristics were identified, indicating a balanced development in logistics matching for the region in those respective years. Furthermore, in 2012 for indicator *X*_*6*_, in 2013 for indicator *X*_*8*_, in 2016 for indicator *X*_*5*_, and in 2017 for indicator *X*_*4*_, there was a significant display of individualized features, with the advantage indicator weights in each case exceeding the critical value of 0.5.

Through an analysis of the weight coefficients associated with individualized characteristics within each logistics region for different years, a correlation is observed between the logistics development stage and the distinctive attributes of the indicators. This insight provides a foundational understanding that can inform and guide the development of strategic improvements in subsequent stages.

#### 2) Matching evaluation analysis

Upon arranging the data, the evaluation rankings are derived that pertain to the individualized characteristics and the matching levels across each logistics region within the period from 2011 to 2020. North China logistics region exhibited individualized characteristics in 2018 but not in 2019. In contrast, Northeast logistics region showed such characteristics in 2019, with a notable absence in 2011. A comprehensive list of these evaluations is provided in Table 41.

**Table 41:** Classification of matching evaluation results in various regions, 2011–2020.

Logistics regions	2011	2012	2013	2014	2015	2016	2017	2018	2019	2020
North China								*	▽	
Northeast	▽								*	
Shandong Peninsula	▽							*		
Yangtze River Delta	*									▽
Southeast Coastal	*	▽								
Pearl River Delta	▽							*		
Central				*						▽
Northwest		▽			*					
Southwest								*	▽	

Note: Rankings should be based on the perspective that is most beneficial to the evaluated object, where

* indicates individualized characteristics and

▽ indicates a lack of individualized characteristics.

Each region can leverage periods when it demonstrated distinctive advantages as a benchmark for enhancing future alignment between logistics architecture and resources. These exemplary years can serve as a blueprint for tailoring subsequent strategies to improve overall coordination. Conversely, periods lacking in distinctive advantages should be scrutinized to uncover the underlying reasons for this shortfall and to identify the contributing factors, thereby preventing similar lapses in the future.

From both the evaluated subject’s perspective and through a democratic evaluation lens, the years when a logistics region’s matching level is high often rank prominently. Instances include Northeast logistics region’s 2019 results and the Yangtze River Delta logistics region’s 2011 results, which were both highly ranked. In contrast, Northwest logistics region experienced a decline in its democratic evaluation ranking for the year 2015, a downturn attributed to poor performance across several key indicators in the assessment.

Using North China logistics region as a case in point, it demonstrated particularly notable individual advantage characteristics in the year 2018. This year can serve as a reference point for future development, allowing the region to build upon and enhance its unique strengths. According to the identification of these distinctive features, North China logistics region particularly excelled in the evaluation index *X*_*6*_ during 2018. Consequently, future development strategies for the region should take into account and aim to maximize the spatial demand advantages that North China logistics region possesses.

In addition, the region was found to be lacking in individual advantage characteristics in the evaluation indices *X*_*4*_ and *X*_*9*_ in 2019. This indicates that there is a need to concentrate future investments on the structure of logistics resources and the timeliness of logistics services. In the same vein, other logistics regions should also utilize the findings from the identification and evaluation of their individual advantage characteristics. This will enable them to devise strategies that are tailored to their specific context, with the goal of achieving sustainable development in regional logistics moving forward.

## IV. Conclusion and discussion

This paper first analyzes the positive impact of the alignment between logistics resources and architecture on the sustainable and eco-friendly development of regional logistics. It introduces an evaluation approach for this alignment, emphasizing the perspective of individual characteristic advantages. The study reveals that sustainable regional logistics development mandates leveraging regional resource strengths to forge a synergistic logistics structure, which in turn reduces logistics expenses and enhances operational efficiency. These findings are in line with current scholarly work.

Subsequently, the paper presents an evaluation index system for the alignment of logistics resources and architecture predicated on the concept of logistics equilibrium. This is augmented by a democratic assessment methodology that highlights individual characteristics. The paper conducts an analysis of the matching level of logistics resources and structure across China’s nine major logistics regions from 2011 to 2020. It identifies the individual characteristics of each region and provides a corresponding democratic evaluation. This approach paves the way for novel strategies in the subsequent research on the green and sustainable progression of regional logistics.

Key findings from the study are as follows,

1. The study observes an overall positive trend in the matching level of logistics resources and structure across different regions in China. However, it also highlights significant regional disparities. Specifically, the Pearl River Delta and Southeast coastal regions have seen robust development, whereas Northeast, Shandong Peninsula, and Northwest regions have lagged in terms of matching logistics development.

2. Each logistics region has demonstrated distinct advantage characteristics during their development. By examining the fluctuating weight coefficients of these characteristics over the years, the study identifies the strengths of each region. This insight is crucial for crafting targeted sustainable development strategies. For instance, North China logistics region can leverage its 2018 advantages as a benchmark or compare itself with leading examples to identify areas for improvement.

3. The research employs a democratic evaluation process to rank and recognize outstanding examples, fostering consensus among the evaluators. This process respects public interests while accentuating the unique strengths of each evaluated entity. Notably, Northeast logistics region in 2019 and the Yangtze River Delta logistics region in 2011 were identified as leading examples based on both the evaluation object’s perspective and the democratic evaluation criteria.

The mainly contributions of this paper are threefold: firstly, it addresses the critical issues of green and sustainable regional logistics development by focusing on the unique advantage characteristics of each logistics region and the harmony between logistics resources and structure, aligning with the current developmental traits and challenges in China’s logistics sector. Secondly, building upon existing research, the paper introduces an index system that not only includes conventional indicators but also integrates the concept of logistics equilibrium, aligning with the principles of green and sustainable logistics development. Thirdly, drawing on the work of Zhao X et al. (2023), this paper constructs a democratic evaluation model that assesses the matching level of logistics resources and architecture based on individual advantage characteristics. This model serves as a valuable tool for devising future strategic directions for regional logistics.

Based on the research conclusions, the following suggestions were proposed:

1. To elevate the development of regional logistics, it is imperative to leverage our unique strengths and to develop distinctive features. According to the research presented, those logistics regions with significant individualized characteristics that foster successful matches are also recognized with higher rankings in democratic evaluations. Consequently, to improve the congruence between logistics architecture and the distribution of resources, there is a need to fully utilize these distinctive traits. Additionally, it is crucial to seek out any absent impact indicators that have the potential to markedly enhance the effectiveness of future matches.

2. To reach an optimal state of congruence, emphasis should be placed on achieving a balanced match within four critical dimensions. The analysis has shown that the logistics regions that have successfully synchronized their logistics architecture and resources—referred to as the "top performers"—demonstrate a balanced distribution of weights across individualized characteristics within the dimensions of quantity, structure, space, and time. Conversely, regions that have obtained lower rankings often exhibit deficiencies in one or several of these dimensions. Therefore, to elevate the alignment between logistics architecture and the distribution of resources, it is imperative to pursue a balanced development strategy that addresses each of the four dimensions equally.

3. Improvements to the match level should be aligned with particular circumstances to ascertain individualized characteristics. Considering that economic levels, resources, policies, and principal social contradictions evolve through different developmental stages, it is essential to formulate unique policies and measures that are attuned to the specific development context and individual traits of each stage. For example, during the unprecedented societal challenges posed by the COVID-19 pandemic in 2020, there was a heightened necessity for expedited and precise logistics delivery. As a result, at this critical period, the logistics architecture and resources ought to have demonstrated distinct individualized characteristics in the dimensions of spatial and temporal matching. Even in instances where there was a shortage of individualized characteristics in quantity matching, the regions were still able to achieve a mid-tier ranking in democratic evaluations. The interplay between the relative optimality of balanced matches across the four dimensions and the manifestation of individualized characteristics under exceptional circumstances is mutually reinforcing

4. The creation of a policy support framework that is aligned with individualized characteristics is both necessary and beneficial. Analysis of the recent trajectory indicates that since 2011, China has been instrumental in formulating and issuing a comprehensive series of policy documents designed to catalyze the high-quality development of the logistics industry, complemented by the execution of corresponding supportive measures. The introduction of these policies has played a pivotal role in the observed improvement of the match level across most logistics regions from 2012 to 2017. The enhancement of this match level is closely associated with the supportive policy environment that has effectively encouraged the development of distinctive features within these regions. As the world progresses into a novel era of development, the ongoing implementation of policies in China that are targeted at sustainable and high-quality logistics development is anticipated to drive the country’s logistics industry towards achieving a new zenith of progress and success.

Utilizing a democratic evaluation approach centered on individual competitive traits, this study conducts an assessment and analysis of the alignment between logistics resources and structure within China’s nine principal logistics regions. The empirical analysis has successfully discerned the distinctive competitive features of logistics across these regions, yielding a collection of significant findings. These insights serve as a reference point for the construction of a sustainable and environmentally friendly logistics framework tailored to the unique strengths of each region. It is important to note, however, that the study’s conclusions are drawn from an analysis grounded in the Chinese context, which may limit their applicability to other nations. In future research endeavors, a comparative analysis of regional logistics across various countries could be undertaken to bolster the reliability and universality of the findings.
